# Pain Perception in Latino vs. Caucasian and Male vs. Female Patients: Is There Really a Difference?

**DOI:** 10.5811/westjem.2017.1.32723

**Published:** 2017-04-17

**Authors:** Molly Aufiero, Holly Stankewicz, Shaila Quazi, Jeanne Jacoby, Jill Stoltzfus

**Affiliations:** * St. Luke’s University Hospital, Department of Emergency Medicine, Bethlehem, Pennsylvania; † Aria Health Network, Department of Emergency Medicine, Philadelphia, Pennsylvania; ‡ Lehigh Valley Health Network, Department of Emergency Medicine, Allentown, Pennsylvania; § St. Luke’s University Hospital, Research institute, Bethlehem, Pennsylvania

## Abstract

**Introduction:**

Pain is a common emergency department (ED) complaint. It is important to understand the differences in pain perception among different ethnic and demographic populations.

**Methods:**

We applied a standardized painful stimulus to Caucasian and Latino adult patients to determine whether the level of pain reported differed depending on ethnicity (N=100; 50 Caucasian [C], 50 Latino [L] patients) and gender (N=100; 59 female, 41 male). Patients had an initial pain score of 0 or 1. A blood pressure cuff was inflated 20 mm HG above the patient’s systolic blood pressure and held for three minutes. Pain scores, using both a 10-cm visual analog scale (VAS) and a five-point Likert scale, were taken at the point of maximal stimulus (2 minutes 50 seconds after inflation), and at one- and two-minute intervals post deflation.

**Results:**

There was a statistically significant difference between the Likert scale scores of Caucasian and Latino patients at 2min 50sec (mean rank: 4.35 [C] vs. 5.75 [L], p<0.01), but not on the VAS (mean value: 2.94 [C] vs. 3.46 [L], p=0.255). Women had a higher perception of pain than males at 2min 50sec on the VAS (mean value: 3.86 [F] vs. 2.24 [M], p<0.0001), and the Likert scale (mean rank: 5.63 [F] vs. 4.21 [M], p<0.01).

**Conclusion:**

Latinos and women report greater pain with a standardized pain stimulus as compared to Caucasians and men.

## INTRODUCTION

Pain is one of the most common complaints in emergency departments (ED) nationwide. The perception of pain in others is, therefore, an important component of patient assessment and treatment. There are difficulties in studying pain since it is subjective, which raises the question of what is a clinically significant change in pain. Todd et al. found that reporting less than a 13 mm change in pain severity on the 100 mm visual analogue scale (VAS) was not clinically significant.^1^

Inequalities in analgesic administration to ED patients of different ethnic and demographic groups have been well documented, but there is limited data on objective differences in pain perception between these ethnic groups or between the two genders. Such differences would be clinically relevant as they could rationally affect the decision to use analgesics and the doses administered. This is especially important today when non-Caucasian minority groups comprise roughly one-third of the U.S. population, a number that is projected to nearly double by the year 2050, according to the U.S. Census Bureau.^2^

The majority of available evidence comparing differences in pain perception between men and women is in agreement. According to a comprehensive literature review published in the *Journal of Pain* in 2009, women have consistently shown a greater sensitivity to pain, both in the clinical and experimental setting.^3^ In one randomized double-blinded study published in *Anesthesia & Analgesia*, researchers sought to electrically induce pain in healthy young subjects to study gender differences in nociception. Cutaneous stimulation of the earlobe allowed measurement of pain detection thresholds and maximal pain tolerance. They found, with statistical significance, that male subjects had greater stimulus thresholds (lower nociception) compared to female subjects, and a greater pain tolerance. 4

The little data available on pain differences among different ethnic groups is conflicting. The studies that are available are heterogeneous in both patient population and methodology, leading to inconclusive evidence. In a 2010 retrospective chart review of approximately 800 patients presenting to a multi-cultural and highly diverse inner-city hospital with a long-bone fracture, examiners sought to determine the differences between self-reported pain scores by ethnic group and English-speaking status. In this study, it was found that pain score did not vary by race, ethnicity or language.^5^ On the contrary, the *Journal of Palliative Care* published a systematic review of the literature in 2014, studying the relationship between ethnicity and the pain experience in cancer patients, and came to a different conclusion. The authors reviewed literature published between 1998 and 2013, included 11 studies, and found that a significantly greater proportion of Hispanics (50%) and Blacks (49%) presented with severe pain at first consultation at a cancer center compared to White (33%) patients. 6, 7 After adjustment for age, sex, stage of cancer, and comorbidities, both Hispanic and Black patients were nearly twice as likely to report severe pain relative to White patients.

The purpose of this prospective study was to better understand the differences in pain perception among our patient population in a community hospital ED. Our objective was to apply a standardized painful stimulus to both Caucasian and Latino patients presenting to the ED to determine whether the level of pain reported and the words used to describe the painful stimulus differed depending on ethnicity and gender. We also sought to examine subjective differences in the manner that pain was described by the different demographic groups.

## METHODS

### Study Setting and Population

This prospective clinical trial was conducted in the two EDs and the medical clinic of our community teaching hospital in a northeastern city in Pennsylvania (combined ED volume of 79,000 patients per year). We enrolled 10% of subjects from the medical clinic. Latino and Caucasian adult patients of both genders (age 18 years and older) who were being seen for a non-painful condition were approached and asked to participate in the study by one of the two investigators. Prior to participating in the study, patients had to have a pain score of 0 or 1. In addition, participants were asked to self-assess their pain tolerance on a 10-point Likert scale, with 1 being “very sensitive to pain,” and 10 being “able to tolerate extreme pain.”

**Population Health Research Capsule**

What do we already know about this issue?

Pain is a common complaint in emergency departments. Inequalities in analgesic administration to ED patients of different ethnic and demographic groups have been well documented.

What was the research question?

Are there objective differences in pain perception among ethnic groups or between the two genders?

What was the major finding of the study?

Latinos and women report greater pain with a standardized pain stimulus as compared to Caucasians and men.

How does this improve population health?

There appears to be a difference in pain perception among ethnic groups and genders. We can improve patient care if we understand the intricacies involved in identifying and treating pain.

### Study Design

**Figure 1:**
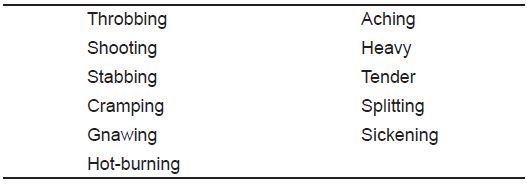
McGill pain descriptors (modified short-form version).

A standard blood pressure cuff, appropriate to the patient’s size, was inflated 20 mm HG above their recorded systolic blood pressure and held for three minutes. Two minutes and 50 seconds after inflation, patients were asked to note their degree of discomfort on a 10 cm VAS and a five-point (0–4) Likert scale. Patients were also queried regarding descriptors of their pain, using those in the short-form McGill Pain Questionnaire (Figure 1). The cuff was then deflated, and at one- and two-minute intervals post deflation the patients repeated the VAS and Likert scale. Consent and survey instruments were available in both English and Spanish.

### Statistical Analysis

Due to the ordinal nature of the VAS, Likert scale and “tolerance” measurement variable, we performed a non-parametric Mann-Whitney (M-W) rank sums test on all data, with results expressed as mean ranks, z-scores and significance values. For comparative purposes only, we re-analyzed the VAS with the independent samples t-test for statistical comparisons of the means between gender and ethnic groups (males and females, Latinos and Caucasians), as these groups’ scores demonstrated normally distributed data.^8^, ^9^ Results of the t-test were analogous to the M-W test (i.e., significant and non-significant outcomes were mirrored). Among the different ethnic and gender groups, the independent samples t-test was also used for comparison of mean values for the McGill pain scale, as the sum total of scores was roughly normally distributed.

## RESULTS

There were 100 subjects, 50 Caucasian (C) and 50 Latino (L) patients who completed the study. Of the 59 female subjects, 28 were Caucasian and 31 were Hispanic. The 41 male subjects included 22 Caucasian males and 19 Hispanic males. The mean age was 43 years; Caucasians were slightly older than Latinos (48 years vs. 39 years, p<0.05), and there was no age difference between genders.

### Caucasians vs. Latinos

**Figure 2:**
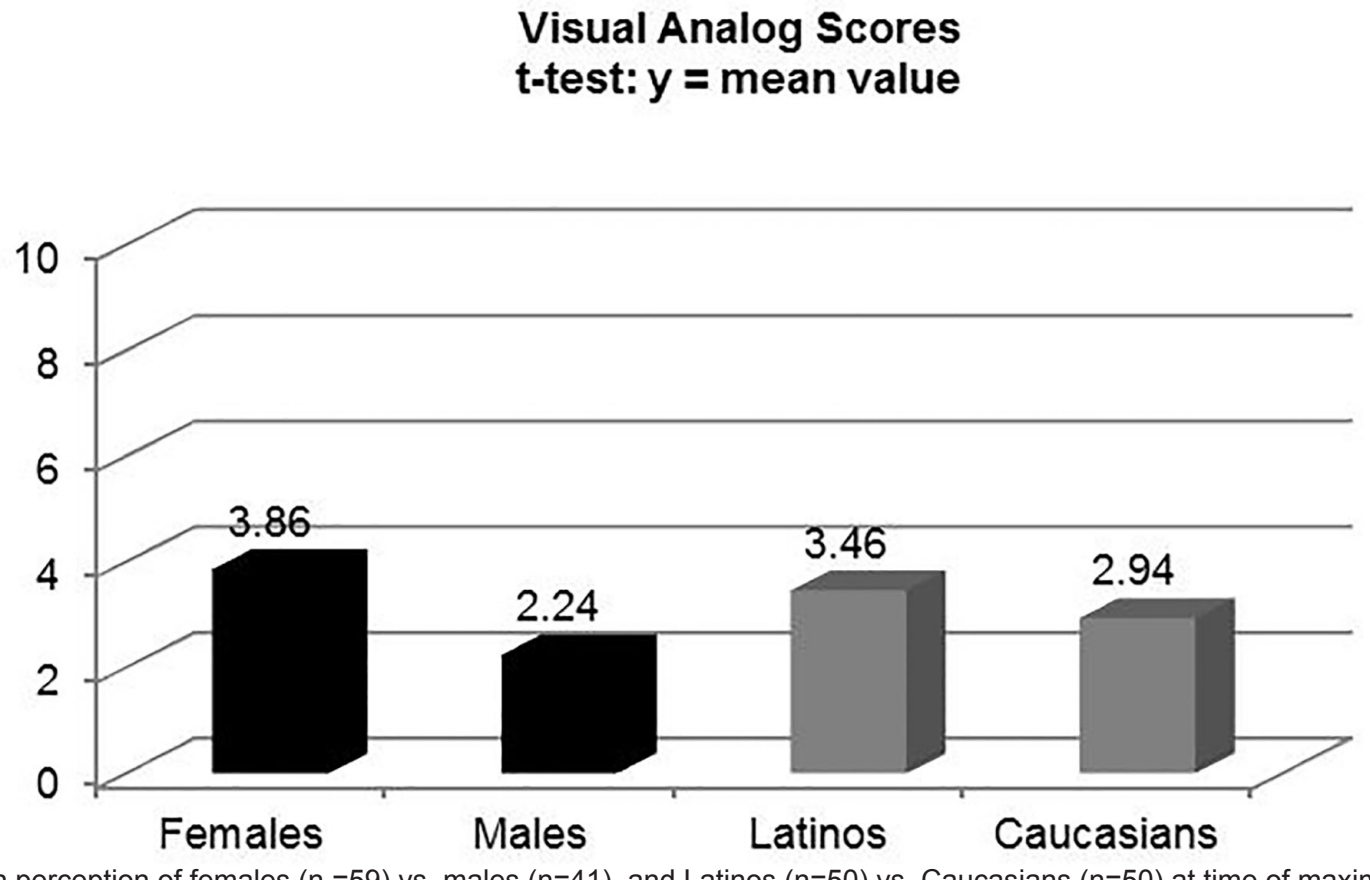
Pain perception of females (n =59) vs. males (n=41), and Latinos (n=50) vs. Caucasians (n=50) at time of maximal painful stimulus (2 min 50 sec) using the 10-cm visual analog scores (VAS). Mean values obtained using the independent samples t-test.

**Figure 3:**
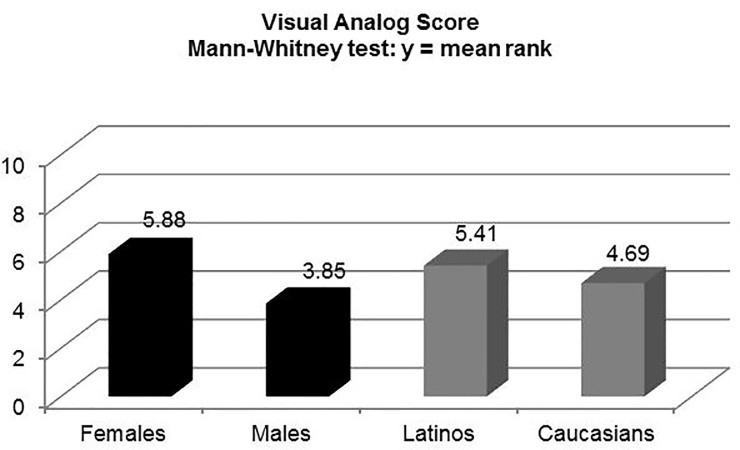
Pain perception of females vs. males and Latinos vs. Caucasians at time of maximal painful stimulus (2 min 50 sec) using the 10-cm visual analog scores (VAS). Mean ranks obtained using the Mann-Whitney rank sums test.

**Figure 4:**
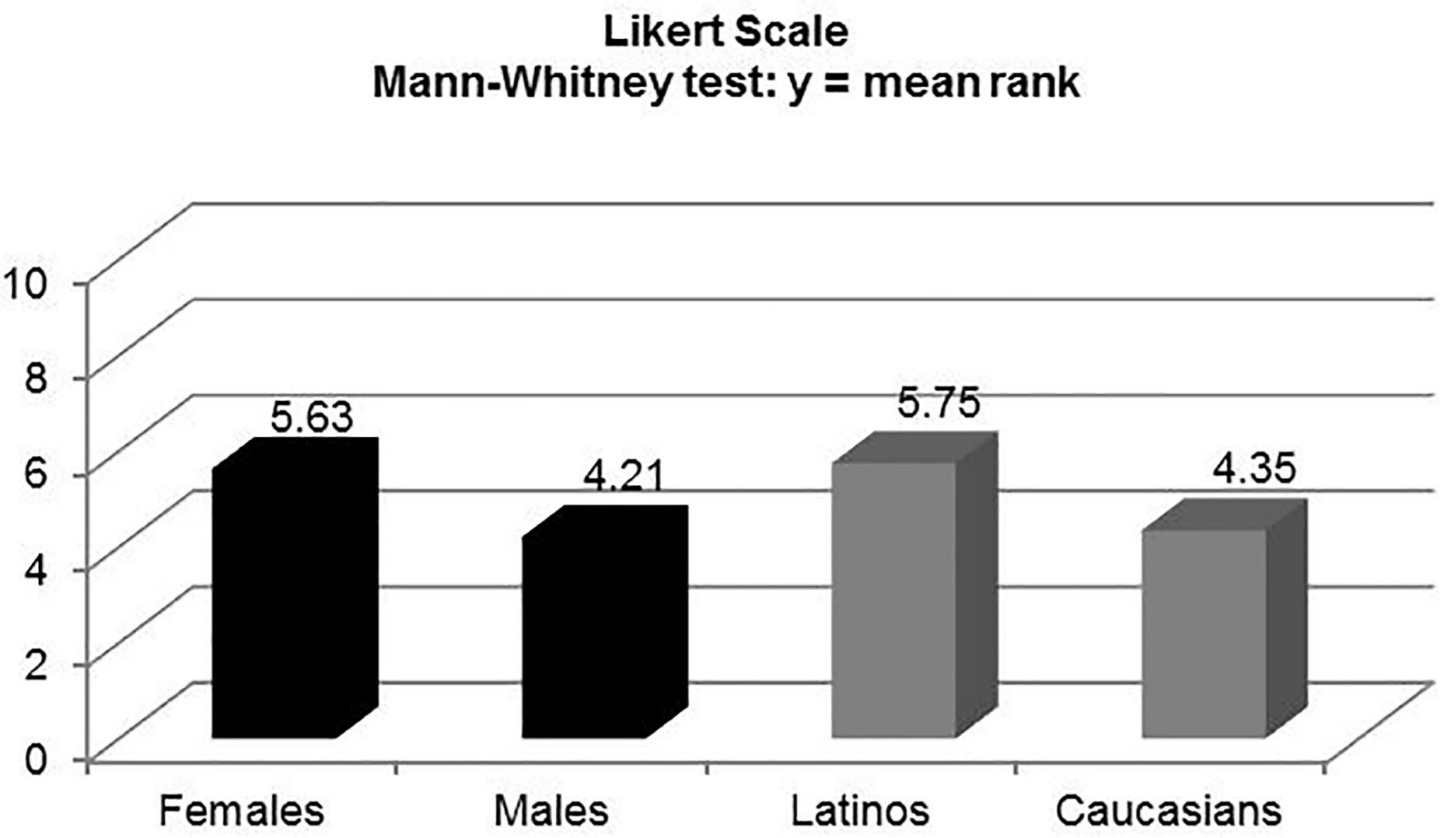
Pain perception of females vs. males and Latinos vs. Caucasians at time of maximal painful stimulus (2 min 50 sec) using the 5-point Likert scale (0–4). Mean ranks obtained using the Mann-Whitney rank sums test.

Caucasians self-reported a higher degree of pain tolerance on the 10-point Likert scale than Latinos, which was statistically significant (M-W mean rank: 4.75 [C] vs. 5.35 [L], p<0.01). There was a statistically significant difference between the five-point Likert scale scores of Caucasian and Latino patients at the time of the maximal painful stimulus, 2min 50sec (M-W mean rank: 4.35 [C] vs. 5.75 [L], p<0.01), but not on the VAS (M-W mean rank: 4.69 [C] vs. 5.41 [L], p<0.211; t-test mean value: 2.94 [C] vs. 3.46 [L], p<0.255) (Figures 2–4). There were no differences in pain perception at one and two minutes post deflation. Perhaps surprisingly, both ethnic groups rated the 11 qualitative McGill pain descriptors almost identically; thus, there was no statistically significant difference (t-test: p=0.18).

### Genders

There were no differences between the two genders in self-assessment of pain tolerance on the 10-point Likert scale (M-W mean rank: 4.99 [M] vs. 5.09 [F], p<0.695). However, the cohort of women (Caucasians and Latinos combined) had a much higher perception of pain than the homologous male cohort at 2min 50sec (time of maximal painful stimulus) on both the VAS (M-W mean rank: 5.88 [F] vs. 3.85 [M], p<0.01; t-test mean value: 3.86 [F] vs. 2.24 [M], 95% confidence interval [CI] [0.78–2.47], p<0.0001), and the Likert scale (M-W mean rank: 5.63 [F] vs. 4.21 [M], p<0.01) (Figures 2–4). There were no statistically significant differences within each gender by ethnicity at the point of maximal stimulus (M-W mean rank: females [2.79 (C) vs. 3.19 (L), p=0.378]; males [1.94 (C) vs. 2.28 (L), p=0.355]). Similarly, there were no differences on either pain scale at one and two minutes post deflation, when the pain had greatly diminished. As was the case with Caucasians vs. Latinos, there was no statistically significant difference between males and females when comparing the mean values for the sum total of categories in the qualitative McGill pain scale (t-test: p=0.26).

## DISCUSSION

In this prospective clinical trial, we found differences in the perception and reporting of pain both between genders and between Caucasians and Latinos. The goal in the ED is to treat the pain of each individual. However, since pain is subjective and often difficult to quantify, the emergency physician may want to consider how different groups have perceived and reported pain tolerance to a standard pain stimulus.

### Caucasians vs. Latinos

Our study demonstrated a statistically significant difference between these two ethnic groups in self-reported pain tolerance. Consistent with this impression, Latinos reported their pain to be significantly greater on the Likert scale and a similar trend (not statistically significant) was noted on the VAS as well. Again, we could not determine the precise cause of this difference, but it did appear that the use of the VAS for Spanish-speaking subjects was less familiar and more difficult to explain than for the Caucasian group.

Ethnic differences in the perception and reporting of pain have occasionally been studied in the past. These studies have shown conflicting results, sometimes indicating no ethnic differences while others suggest African-American and Hispanic patients perceive and report more pain compared to Caucasians.^10^–^12^ Again, the studies are extremely heterogeneous in patient population and methodology. There is also some evidence that the physician’s perception of whether a patient is exaggerating symptoms was associated with the patient’s ethnicity.^13^

### Genders

Unlike the ethnicity-related results, men and women did not differ in the degree to which they assessed their own pain tolerance. However, our results clearly showed a clinically and statistically significant difference in the reported perception of pain by women as compared to men. Similar results have been reported in most but not all of previous studies.^14^–^17^ Differences in study design and statistical methods may well explain why some studies had different results than our study: subjects in earlier trials were either normal volunteers or patients with chronic pain and were not limited to ED patients.

Women consistently demonstrate a trend towards being more sensitive to pain and higher expressions of pain intensity as compared with men; little data has been reported that show women to be more tolerant of pain than men.^14^–^17^ Although the reasons for these gender differences are unclear, speculation has included inherent and acquired differences in emotionality and communication. Factors such as the gender of the experimenter (both investigators in the current study were female), the location of pain, and the type of scale used have also been hypothesized.

## LIMITATIONS

Compared to previous investigations, the current study has some significant limitations as well as several unique strengths. First, we acknowledge a modest sample size of 50 in each ethnic group, which might hinder our ability to detect a true difference in patient response (a type B error). Secondly, the two investigators were not Spanish-speaking and that may have affected the accuracy of some patient responses despite the fact that the study instrument was printed in Spanish and English. Different blood pressures may have also influenced results because if the subject had a baseline higher blood pressure, then the cuff would have been inflated more. Subjects who frequently have their blood pressure taken may be more tolerant of this painful stimulus. In addition, comorbid conditions such as diabetes or peripheral vascular disease may have affected sensory perception, and medications such as calcium channel blockers may likewise have affected sensory perception. Chronic pain syndromes and use of chronic pain medication may also influence pain perception. Finally, the degree of pain caused by our stimulus was modest, as seen on both the VAS and Likert scale. Use of a more noxious stimulus might elicit different responses. Strengths of our study include the fact that we enrolled equal numbers of patients representing the two ethnicities, all patients presented to a community hospital ED or medical clinic, and all study participants were subjected to a standardized stimulus.

Future studies may be able to more accurately assess pain perception among various ethnic and demographic groups by blinding study participants to the fact that their pain score is being studied. A retrospective study and chart review is currently being planned at our institution. By blinding study participants, we can effectively eliminate observer bias and/or Hawthorne effect. After validating that there is in fact a difference in pain perception among ethnic and demographic groups, the next step is to understand why these differences exist and to correlate these differences to the inequalities that exist in analgesic administration. If we can fully understand the intricacies involved in identifying and treating pain, we can ultimately improve patient care.

## CONCLUSION

Latinos and women report greater pain with a standardized pain stimulus as compared to Caucasians and men. Both genders and ethnicities use similar terms to qualitatively describe the painful stimulus. Future studies are needed to evaluate if these differences exist when patients are blinded to that fact that their pain score is being studied.

## References

[1] Todd K, Funk K, Funk J (1996). Clinical significance of reported changes in pain severity. Ann Emerg Med.

[2] The United States Census Bureau https://www.census.gov/en.html.

[3] Fillingim R, King C, Ribeiro-Dasilva M (2009). Sex, Gender, and Pain: A Review of Recent Clinical and Experimental Findings. J Pain.

[4] Walker S, Carmody J (1998). Experimental pain in healthy human subjects: gender differences in nociception and in response to ibuprofen. Anesth Analg.

[5] Bautista F, Mejia JG, Garg N (2010). Self-Reported pain scores are not affected by language, race and ethnicity among adults presenting to the emergency department with long bone fractures. Ann Emerg Med.

[6] Reyes-Gibby CC, Anderson KO, Shete S (2012). Early referral to supportive care specialists for symptom burden in lung cancer patients: a comparison of non-Hispanic whites, Hispanics, and non-Hispanic blacks. Cancer.

[7] Kwok W, Bhuvanakrishna T (2014). The relationship between ethnicity and the pain experience of cancer patients: a systematic review. Indian J Palliat Care.

[8] Dexter F, Chestnut DH (1995). Analysis of statistical tests to compare visual analog scale measurements among groups. Anesthesiology.

[9] Mantha S, Thisted R, Foss J (1993). A proposal to use confidence Intervals for visual analog scale data for pain measurement to determine clinical significance. Anesth Analg.

[10] Todd K, Samaroo N, Hoffman J (1993). Ethnicity as a risk factor for inadequate emergency department analgesia. JAMA.

[11] Todd K, Lee T, Hoffman J (1994). The effect of ethnicity on physician estimates of pain severity in patients with isolated extremity trauma. JAMA.

[12] Edwards R, Fillingim R (1999). Ethnic differences in thermal pain responses. Psychosom Med.

[13] Miner J, Biros M, Trainor A (2006). Patient and Physician perceptions as risk factors for oligoanalgesia: a prospective observational study of the relief of pain in the emergency department. Acad Emerg Med.

[14] Mylius V, Kunz M, Schepelmann K (2005). Sex differences in nociceptive withdrawal reflex and pain perception. Somatosens Mot Res.

[15] Soetanto A, Chung J, Wong T (2006). Are there gender differences in pain perception?. J Neurosci Nurs.

[16] Nevin K (1996). Influence of sex on pain assessment and management. Ann Emerg Med.

[17] Blomkalns A, Kalokhe G, Lindsell C (2005). Sex differences in pain perception and description. Acad Emerg Med.

